# Intelligent Control Method for Loss Distribution Balance of High-Power Photovoltaic Grid-Connected Inverters

**DOI:** 10.1155/2022/7240834

**Published:** 2022-05-31

**Authors:** Yi Xu, ErYong Zou, FengPing Tang

**Affiliations:** ^1^Jiaxing Nanyang Polytechnic Institute, Jiaxing, Zhejiang 314000, China; ^2^Key Laboratory of Control of Power Transmission and Conversion (SJTU), Ministry of Education, Shanghai 200240, China

## Abstract

Aiming at the problem that the loss distribution balance control effect of high-power photovoltaic grid-connected inverter is poor due to the complex loss factors, this paper proposes a loss distribution analysis and balance intelligent control method for high-power photovoltaic grid-connected inverter. The topology structure of high-power photovoltaic grid-connected inverter is constructed and the overall control scheme is designed. The loss of inductance, resonant frequency, harmonic attenuation, and damping resistance in the circuit of PV grid-connected inverter is analyzed, respectively. On this basis, a two-stage loss control model of high-power PV grid-connected inverter is constructed, and the generalized linear decision rule is adopted to schedule PV power supply and fluctuating load. A two-stage loss control model for high-power photovoltaic grid-connected inverter was established and the optimal loss control value was obtained. Experimental results show that the proposed method can accurately suppress voltage and current losses of grid-connected photovoltaic inverters and has strong resonance resistance and robustness.

## 1. Introduction

In recent years, photovoltaic power generation has accounted for an increasing proportion of the installed capacity in the power system. As a kind of distributed generation, photovoltaic power generation has also been widely studied. According to different needs and applications, photovoltaic grid-connected power generation systems have different structures and power levels, from a single photovoltaic module to a million-level photovoltaic module [1]. With the continuous improvement of requirements for grid-connected voltage level, efficiency, and grid-connected current quality of photovoltaic grid connected inverter, multilevel photovoltaic grid-connected inverter is bred [2–4]. The multilevel conversion technology can avoid the direct series connection of power switching devices. It has the advantages of high output voltage, low harmonic content, small switching stress, low voltage change rate, and low switching frequency. It can be applied to the fields of medium voltage high-power AC speed regulation, high-voltage DC transmission, static var generator, and new energy power generation [5–7]. When the large-scale photovoltaic power generation system is connected to the grid, the impact on the power quality of the power grid and the operation conditions such as the separation speed of the power grid in case of failure of the photovoltaic power generation system itself has become an important focus [8]. The research on the intelligent control method of loss distribution balance of high-power photovoltaic grid-connected inverter has become the main research content in the current field.

At present, relevant scholars have made research on the loss distribution of high-power photovoltaic grid-connected inverters and proposed an intelligent control method for the balance of loss distribution of photovoltaic grid-connected inverters. Reference [9] presents a control method for a two-stage single-phase grid-connected cascaded H-bridge photovoltaic inverter based on a hybrid control strategy of optimal third harmonic injection and limited power maximum power point tracking. The optimal third harmonic injection can improve the modulation range; limited-power maximum-power point tracking algorithm can limit the power of the photovoltaic module of the unit where the maximum modulation ratio is located under the condition of exceeding the third harmonic compensation range, so that it can continue to meet the grid-connected operating conditions. Reference [10] proposes a direct power control method based on nonlinear disturbance observer. In the photovoltaic grid-connected power generation system, when the output power of photovoltaic panels suddenly changes, the DC side bus voltage fluctuates greatly. If the control is improper, it will directly affect the normal operation of the system. This method does not need to add additional sensors. The disturbance observer is used to observe the output power of the photovoltaic panel in real time, and it is added to the inner power loop as a feedforward value, so that the given power inner loop contains the input power information. For the power inner loop, the control input is redefined, and the synchronous rotation coordinate transformation is omitted. The design process of the observer and the power inner loop is introduced in detail, and the parameter tuning method of the inner and outer loops is given.

The above methods all provide a certain theoretical support for the control of high-power photovoltaic grid-connected inverters, but the above methods do not fully consider the loss distribution of high-power photovoltaic grid-connected inverters, and the control effect on the loss of photovoltaic grid-connected inverters is relatively low. Difference. For this reason, this paper proposes an intelligent control method for the loss distribution balance of high-power photovoltaic grid-connected inverters, fully analyzes the inductance, resonant frequency, harmonic attenuation, and damping resistance losses in the photovoltaic grid-connected inverter circuit, and a two-stage loss control model is proposed, and the intelligent control of loss distribution balance of photovoltaic grid connected inverter is realized.

## 2. Topology Structure and Overall Control Scheme of High-Power Photovoltaic Grid-Connected Inverter

### 2.1. Topology

**Figure 1:**
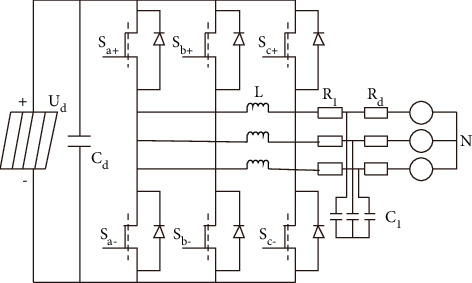
Topological structure of high-power photovoltaic grid-connected inverter.

The function of the high-power photovoltaic grid-connected inverter is to convert the DC power output by the high-power photovoltaic panels into the AC power of the same frequency and phase of the large grid voltage. In order to study the intelligent control method of the loss distribution balance of the high-power photovoltaic grid-connected inverter, this paper chooses a typical two-stage high-power photovoltaic grid-connected inverter topology; its topology is shown in Figure 1.

As shown in Figure 1, *U*_*d*_ represents the output voltage of the high-power photovoltaic array; *C*_*d*_ represents the filter capacitor on the input side; (*S*_*a*+_,*S*_*a*−_), (*S*_*b*+_, *S*_*b*−_), and (*S*_c+_, *S*_c−_) represent the switch tubes that constitute the key components of the inverter; *R*_*l*_ represents the missed filter *R*_*d*_ represents the grid resistance of the large grid; *C*_*l*_ represents the inverter capacitance; *N* represents the neutral point of the large grid, which is used as the potential reference point [11, 12]. The core of the topology structure of the high-power photovoltaic grid-connected inverter is the realization of the control theory, so the requirements for the topology structure of the high-power photovoltaic grid-connected inverter are relatively high. The voltage space vector control which is easy to digitize is adopted, which has the effect of improving the utilization rate of DC voltage and has the characteristics of many voltage steps of output voltage, so it has small voltage harmonic component, meeting the needs of topology design of high-power photovoltaic grid-connected inverter.

In order to construct the mathematical model of the high-power photovoltaic grid-connected inverter, its switching function is defined [13], as shown in the following formula:

(1) $$\begin{array}{c} \left\{\begin{array}{c} d_{1}=\left\{\begin{array}{cc} 1\;S_{a+} & \text{turn on}\\ -1\;S_{a-} & \text{shut down} \end{array}\right)\\ d_{2}=\left\{\begin{array}{cc} 1\;S_{b+} & \text{turn on}\\ -1\;S_{b-} & \text{shut down} \end{array}\right)\\ d_{3}=\left\{\begin{array}{cc} 1\;S_{c+} & \text{turn on}\\ -1\;S_{c-} & \text{shut down} \end{array}\right) \end{array}\right). \end{array}$$

In formula (1), *S*_*a*+_, *S*_*b*+_, and *S*_*c*+_ represent the inverter coefficient in the open state, and *S*_*a*−_, *S*_*b*−_, and *S*_*c*−_ represent the inverter coefficient in the closed state. The inverter output voltage is the voltage between the inverter output phase and the neutral point plus the neutral point voltage [14]. According to the balance theory and the characteristics of capacitance and inductance on voltage and current, the mathematical model of high-power photovoltaic grid-connected inverter is derived as

(2) $$\begin{array}{c} \dot{x}=Ax+Bi. \end{array}$$

In formula (2), $\dot{x}$ represents the phase voltage of the inverter output filter; *x* represents the large grid voltage; *A* and *B* represent the coefficient matrix; *i* represents the inverter output filter current.

### 2.2. Overall Control Scheme of Photovoltaic Inverter

**Figure 2:**
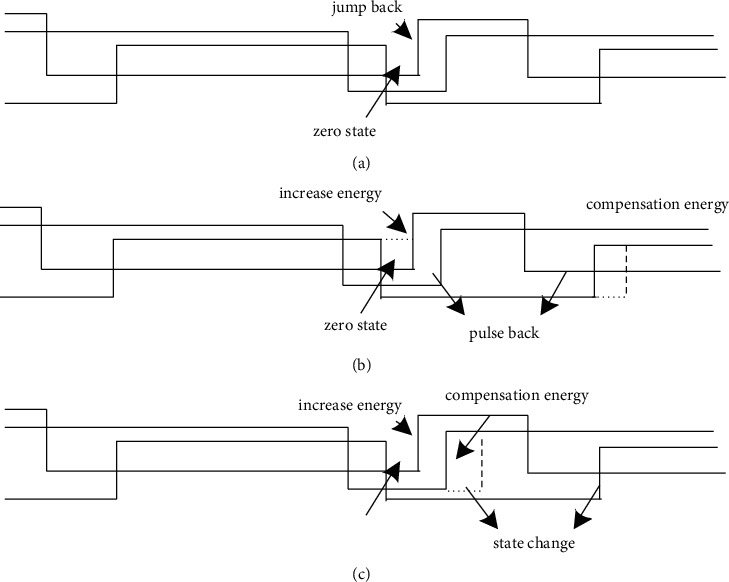
The translation process of the carrier pulse of photovoltaic inverter (Jump backward), (Pulse backward), and (State change).

The adoption of the photovoltaic inverter carrier phase-shift control strategy can avoid the state where the voltage of the photovoltaic inverter integrated circuit is zero, so that the modulation index *n* > 0.462 in the phase-shift control system can be optimized in the photovoltaic inverter optimization control system to stabilize the control modulation area below 0.462 [15]. Under such conditions, the DC voltage *u*_*b*_ of the photovoltaic inverter integrated circuit drops to 1. If the photovoltaic inverter carrier phase-shift control scheme needs to be adopted in the area of *n* < 0.462, the current in each circuit will have a certain peak value, which reduces the utilization rate of photovoltaic inverters [16, 17]. When *n* is the domestic standard value of 0.5, the carrier amplitude of the three output terminals of the PV inverter carrier phase-shift control is 2 V, the period is *T*_2_, the carrier signal reaches the peak value, the sine wave first falls and then rises, and the number of carrier switches increases to 2. Second, the zero state is in a small variation range and lasts for a short time. So the photovoltaic inverter always works under the current light and temperature. In the process of optimizing the modulation strategy of the modulation control system, the level of the differential mode filter supplements the energy in the back-shifted state and is equivalently exchanged with the zero-state voltage of the circuit, thereby effectively improving the mutation of the phase-shifted carrier and maintaining the switching. The number of times does not change. The low-level state appearing in the pulse transformation is regarded as the zero state of the integrated circuit, which is equivalent to the carrier pulse transformation in the equivalent state [18–20]. The carrier pulse translation process is shown in Figure 2.

According to Figure 2, it can be seen that if the pulse signal sine wave is distorted at the intersection of the rectangular wave, the duration of the zero state of the circuit will be prolonged. When the potential of the intersection of the triangular wave is reduced to the lowest point, the duration of the zero state of the integrated circuit is significantly shortened, and the high level jumps. The trigger pulse signal is variable, so when setting the zero state of the photovoltaic inverter integrated circuit, the change of the pulse width causes the output voltage to be uneven to a certain extent, so that the differential mode properties of the pulse are frequently switched, and it needs to be compensated in the shortest time because the voltage bump changes the pulse energy, the pulse modulation strategy of the photovoltaic inverter changes the position of jump and shift, and the number of pulse switches of the photovoltaic inverter is retained [21].

## 3. Loss Distribution of High-Power Photovoltaic Grid-Connected Inverters

### 3.1. Basic Current Formula

Since the loss of the switching device has a direct relationship with the current flowing through it, the relationship between the voltage and current of the intermediate bus in the circuit is deduced [22], and the influence of the photovoltaic grid-connected inverter on the current is deduced according to the inverter harmonic circuit model.

(3) $$\begin{array}{c} G_{n}=\frac{I_{A-n}\left(s\right)}{U_{A-n}\left(s\right)}. \end{array}$$

In the above formula, *G*_*n*_ represents the influence of the photovoltaic grid-connected inverter on the current; *I*_*A*−*n*_(*s*) represents the phase current harmonic under the *n* harmonic; *U*_*A*−*n*_(*s*) represents the phase voltage harmonic of the grid under the *n* harmonic.

To sum up, as the harmonics of photovoltaic grid-connected inverters increase, the current harmonics increase rapidly. It can be seen that the quality of photovoltaic grid-connected inverter is extremely sensitive to inverter harmonics, which will cause the power supply quality to fail to meet user needs [23].

### 3.2. Loss Analysis of Photovoltaic Grid-Connected Inverters

#### 3.2.1. Inductance

According to the L-type filter standard, the total inductance is analyzed for the suppression of harmonics in the low input voltage inverter. According to the grid-connected standard, the harmonic amplitude of each order current in the inverter is set to *i*_*g*_(*n*), and the filter is calculated by the following formula. The inductance value of the device *Z* is

(4) $$\begin{array}{c} Z=\max\left[\frac{u\left(n\right)}{n\times \theta_{s0}\times i_{g}\left(n\right)}\right]. \end{array}$$

In the above formula, *n*=2,3,4 and *θ*_*s*0_ represents the fundamental angular frequency; *u*(*n*) represents the harmonic amplitude of each order phase voltage output in the inverter.

When calculating the current harmonic amplitude *i*_*g*_(*n*) of each order, the parasitic resistance existing in the inductor is not considered:

(5) $$\begin{array}{c} i_{g}\left(n\right)=\frac{u\left(n\right)}{\theta_{s0}\times \left[\left(Z_{1}+Z_{2}\right)-Z_{1}Z_{2}\right]}. \end{array}$$

In the above formula, *Z*_1_ and *Z*_2_ represent the grid-side inductance and the inverter-side inductance, respectively.

On the basis of the above formula, the inductive loss value *Z*_eq_ of the *n*-th current harmonic output in the LCL filter is obtained:

(6) $$\begin{array}{c} Z_{eq}=\left(Z_{1}+Z_{2}\right)\times \left(1-\frac{n\theta_{s0}}{\theta_{res}}\right). \end{array}$$

In the above formula, *θ*_res_ represents the resonant angular frequency.

#### 3.2.2. Resonant Frequency

When resonance occurs, the LCL filter with zero impedance resonance point will affect the stability of the power grid control system, resulting in a decrease in power quality and distortion of the output current [24].

Regardless of the parasitic resistance existing in the inductance, the parallel branch of inductance *Z*_1_ on the series inductance side, the inverter inductance *Z*_2_, and capacitor *C*, for the midpoint of the bridge arm, the voltage transfer impedance *X*_1_ generated by the output current of the inverter can be calculated by the following formula:

(7) $$\begin{array}{c} X_{1}=j\theta \left(Z_{1}+Z_{2}-C\theta^{2}\right). \end{array}$$

Set the inverter inductance *Z*_2_ to zero, and calculate the resonant frequency *g* and the resonant corner frequency *θ* by

(8) $$\begin{array}{c} \left\{\begin{array}{c} g=\frac{1}{2\pi }C\sqrt{\frac{Z_{1}+Z_{2}}{Z_{1}Z_{2}}},\\ \theta =C\sqrt{\frac{Z_{1}+Z_{2}}{Z_{1}Z_{2}}}, \end{array}\right) \end{array}$$

#### 3.2.3. Harmonic Attenuation

According to the relationship between the output current and the input current of the LCL filter, the current harmonic attenuation of the filter is designed, and the relationship between the output current *i*_0_ and the input current *i*_*i*_ is described by the following transfer function *H*_*i*_(*s*) combined with the topology of the filter:

(9) $$\begin{array}{c} H_{i}\left(s\right)=\frac{i_{0}\left(s\right)}{i_{i}\left(s\right)}. \end{array}$$

Express the above transfer function *H*_*i*_(*s*) in current form:

(10) $$\begin{array}{c} i\left(n\right)=i_{0}\left(n\right)\left|1-\left(\frac{n\theta_{0}}{\theta }\frac{Z_{1}+Z_{2}}{Z_{1}}\right)\right|. \end{array}$$

Solve the above equation to obtain the corner frequency *g*′ of the LCL filter:

(11) $$\begin{array}{c} g\prime =\frac{1}{2\pi }\sqrt{\frac{2}{Z_{2}C}}. \end{array}$$

If the corner frequency *g*′ is lower than the harmonic current frequency existing in the output current of the low input voltage inverter, after processing by the LCL filter, attenuation will occur. Therefore, the current harmonics whose frequency is higher than the turning frequency have higher suppression ability. However, when the LCL filter is used to process the current harmonics whose frequency is lower than the corner frequency *g*′, it is easy to reduce the waveform quality and cause the waveform to be distorted. Therefore, when designing the harmonic attenuation of the LCL filter, it is necessary to reduce the corner frequency *g*′.

#### 3.2.4. Damping Resistor

In order to avoid resonance in the low input voltage converter, it is necessary to introduce a damping resistor to improve the stability. At the same time, it is necessary to consider the influence of the damping resistor and the stability requirements. Adding a damping resistor to the inverter will increase its power loss. By reducing the resistance value of the damping resistor to reduce the power loss of the low input voltage inverter, the resonance suppression effect is not ideal at this time; increasing the resistance value of the damping resistor in the inverter can improve the stability of the inverter, but, at the same time, it will increase the power loss, which will affect the bypass effect of high-order current harmonics. When suppressing high-frequency harmonics, the ability of the LCL filter is reduced [25].

## 4. Loss Control of High-Power Photovoltaic Grid-Connected Inverters

### 4.1. Construction of a Two-Stage Loss Control Model for High-Power Photovoltaic Grid-Connected Inverter Losses

The front-stage DC-DC conversion of the photovoltaic power generation inverter is to track the control of the maximum power. The power output of the photovoltaic cell has a maximum value and a minimum value. By tracking the power change, the required maximum power is output. Under different temperature and illumination conditions, the power of photovoltaic power generation will be affected differently, so the tracking of the maximum power is necessary for the research and control of the utilization rate of the photovoltaic power generation system and the equipment.

The poststage AC converter stabilizes the DC bus voltage between the front and rear stages to achieve the balance between the output power of photovoltaic power generation, the input power of the grid, and the power of the energy storage device; to control the output current of the system; and to control the waveform. The quality is optimized so that the output current can track the voltage of the grid.

In order to make the system reach the maximum power point voltage as soon as possible and avoid the power loss caused by the system searching for the area far from the maximum power point during the startup process, the compound MPPT method of constant voltage tracking method and disturbance observation method is adopted.

In addition, referring to the idea of intermittent scanning method, the photovoltaic array periodically changes the array voltage and works at this point. Since the actual situation is that the operating point of the array does not change much in a short period of time during the operation of the day, the system uses the method of starting MPPT every 500ms. This method does not require the grid-connected inverter to be in the search state all the time, does not generate oscillation, and avoids the power loss caused by other schemes due to the need for real-time search, and it has proved to have almost no impact on accuracy.

The system has the problem of cooperation between MEPT and MPPT. In the stable stage, MEPT is embedded in MPPT due to the large difference in the disturbance time between the two. Different from the ordinary disturbance observation method, in order to make the system perform MEPT as soon as possible in the startup phase to achieve the maximum efficiency, this paper adds a step change counter in the system startup phase; that is, after each disturbance, it is judged whether the disturbance step size has changed. When a change occurs, the counter is incremented by 1. If the step size changes for the first time, it means that the photovoltaic array operating point has crossed the vertex of the photovoltaic curve; when the second step size changes, it means that the operating point has completed crossing the vertex of the photovoltaic curve twice, and the next beat works. The point will move towards the apex, at which time the array must work near the apex of the photovoltaic curve and oscillate near the apex. The value of the counter is equal to 2, wait for one beat, and trigger the start of MEPT. After the system startup process is over, the design method is the same as the ordinary disturbance observation method.

#### 4.1.1. Calculation of Two-Stage Loss Control Objective Function

Aiming at minimizing the losses of photovoltaic grid-connected inverters, the two-stage loss control objective function is calculated. It is determined that, during the operation of the distribution network, its reactive power is compensated locally, and the structure does not change. Only the optimal scheduling of active power is taken into account, and the photovoltaic power supply and fluctuating load are regarded as the uncertain random variables of loss control. An uncertainty set contains all uncertain random variables in the fluctuation range [26]. In the first stage of optimal scheduling, the predicted values of uncertain random variables, that is, the active power of photovoltaic power sources and loads, are selected as the decision variables of this stage. There is a certain error between the predicted value and the actual value, and the actual value is unknown [27]. The first-stage loss control objective function is

(12) $$\begin{array}{c} C_{1}=\sum_{t=1}^{N}\left(U_{t}+A_{t}+B_{t}+R_{t}\right)t. \end{array}$$

In the above formula, *C*_1_ is the schedulable operation cost of the first stage, *N* is the loss control scheduling cycle, *U*_*t*_ is the photovoltaic energy scheduling cost at *t* time, *A*_*t*_ is the fluctuating load scheduling cost at time *t*, *B*_*t*_ is the energy storage scheduling cost, and *R*_*t*_ is the distribution network loss cost [28]. In the second stage of optimal dispatching, the load loss and light rejection are taken as decision variables. At this time, the actual value of the uncertain random variable is known, and its loss control objective function is

(13) $$\begin{array}{c} C_{2}=\sum_{t=1}^{N}\left(H_{t}+K_{t}\right). \end{array}$$

In the above formula, *C*_2_ is the schedulable operation cost of the second stage, *H*_*t*_ is the reduced penalty cost of light abandonment, and *K*_*t*_ is the reduced penalty cost of load loss. Taking formulas (12) and (13) as the objective functions of loss control, the random variables in the uncertain set can also minimize the total expected cost of the distribution network in the worst case. So far, the calculation of the two-stage loss control objective function has been completed.

#### 4.1.2. Calculation of Two-Stage Loss Control Constraints

When the random variable in the uncertain set takes any value, calculate the various constraints contained in it, obtain the extreme bad scene of the uncertain random variable, and optimize the objective function. The expression of the first-stage loss control equation constraint is

(14) $$\begin{array}{c} \left\{\begin{array}{c} P=P_{1}+P_{2}+P_{3}-P_{4}-P_{5},\\ \frac{P^{2}+Q^{2}}{V^{2}}=I^{2}. \end{array}\right) \end{array}$$

In the above formula, *P* is the power flow distribution, *P*_1_ is the predicted photovoltaic power output value, *P*_2_ is the maximum fluctuation difference of the active power output value, *P*_3_ and *P*_4_ are the charging and discharging capacities of the distribution network, *P*_5_ is the load active power, and *V* is the active power of the branch; *Q* and *I* are circuit resistance and current, respectively [29–31]. By formula (15), the branch power flow constraint is carried out on the objective function of the first stage. According to the electricity conservation of the distribution network and the limit of charging and discharging power, the loss control is constrained by inequality, and the expression formula is

(15) $$\begin{array}{c} \left\{\begin{array}{c} P_{\min}\leq P_{1}\leq P_{\max},\\ S_{\min}\leq S_{1}-S_{2}\leq S_{\max}. \end{array}\right) \end{array}$$

In the above formula, *P*_min_ and *P*_max_ are the lower limit and upper limit of the active power of the photovoltaic power supply, *S*_min_ and *S*_max_ are the lower limit of the discharging power and the upper limit of the charging power, and *S*_1_ and *S*_2_ are the charging power and the discharging power, respectively [32]. The first stage of loss control is used as the reference value to determine the fluctuation range of photovoltaic power output and load. The second stage adopts distributed loss control theory to further reduce the fluctuation of photovoltaic output and load. It provides reactive auxiliary services [33]. Since this process will produce abandoned light, the PV inverter reactive power auxiliary constraint is carried out for the second stage, and the expression is

(16) $$\begin{array}{c} F=\frac{\lambda }{G}. \end{array}$$

In the above formula, *F* is the apparent power of the photovoltaic inverter, *λ* is the binary variable during reactive auxiliary service, and *G* is the maximum power of the access node. According to the recourse cost, the second-stage loss control is constrained by inequality, and the expression is

(17) $$\begin{array}{c} \left\{\begin{array}{c} 0\leq \frac{D}{a}\leq D_{\max},\\ 0\leq \frac{V}{b}\leq V_{\max}. \end{array}\right) \end{array}$$

In the above formula, *V* is the loss of load, *V*_max_ is the maximum loss of the loss of load, *D* is the amount of abandoned light, *D*_max_ is the maximum loss of the amount of abandoned light, and *a* and *b* are the penalty coefficients of the loss of load and the amount of abandoned light, respectively [34]. Traverse all the nodes connected to the photovoltaic inverter, set the voltage of node *j* to *U*_*j*_, set the maximum voltage and the minimum voltage of the node to *U*_max_ and *U*_min_, respectively, and impose voltage constraints on the uncertain set. The formula is

(18) $$\begin{array}{c} U_{\min}\leq \sum_{j=1}^{\delta }U_{j}\leq U_{\max}. \end{array}$$

In the above formula, *δ* is the number of nodes connected to the PV inverter. Through the objective function and constraints, the loss control scheduling results under the worst distribution of uncertain random variables are expressed. At this point, the calculation of the constraints is completed and the model construction is realized.

### 4.2. Solving the Two-Stage Loss Control Model of High-Power Photovoltaic Grid-Connected Inverter Losses

Using generalized linear decision rules, the two-stage loss control model of high-power photovoltaic grid-connected inverter losses is solved, and photovoltaic power sources and fluctuating loads are dispatched. The principles followed in the process of establishing the model are as follows. The first is the simplification principle: the actual model is a multivariable and multilevel complex model, and the establishment of the model requires the necessary simplification of the prototype. Therefore, the model establishment process should be the “simplest” model. The second is the derivation principle: a series of definite conclusions can be deduced through the study of the model. If the model cannot be mathematically deduced and the result of the application prototype cannot be determined, the model will be meaningless. The third is the similarity principle: the model is a form of numerical expression, which needs to have “similarity” with the actual prototype. The key to modeling is to reasonably use the mathematical formula and analytical graphics “similar” to the prototype. The established environment should use new independent variables or existing variables as environment settings. An auxiliary variable is introduced to simulate the change of an uncertain random variable through an affine function to obtain the probability distribution information of the random variable [35]. The calculation formula of auxiliary variable *e* is

(19) $$\begin{array}{c} e=\frac{f\left(v\right)}{E}. \end{array}$$

In the above formula, *v* is the random variable in the uncertain set, *f*(*v*) is the affine function of the random variable, and *E* is the random value function of the random variable. The loss control model is solved recursively. Through mixed-integer linear programming, *e* is transformed into the first-stage auxiliary variable *o*_1_ and the second-stage auxiliary variable *o*_2_. The transformation formula is

(20) $$\begin{array}{c} \left\{\begin{array}{c} o_{1}=\frac{\left(y_{1}-y_{2}\right)cp}{h_{\text{max}}-h_{\text{min}}},\\ o_{2}=q\left(e-o_{1}\right). \end{array}\right) \end{array}$$

In the above formula, *y*_1_ are *y*_2_ are the adjustment power of the photovoltaic inverter reactive power and active power, *c* is the correction coefficient, *p* is the power of the abandoned light, *q* is the compensation capacity of the inverter access node, and *h* is the compensation capacitor. Through auxiliary variables, a fuzzy upper bound is applied to the objective function of the two stages, and the global optimal value in the worst scenario is obtained, the value of the uncertain random variable is continuously updated iteratively, and the set of decision variables which satisfies the expected cost minimization is selected to obtain optimal decisions for two-stage loss control scheduling [36]. So far, the solution of the two-stage loss control model of the high-power photovoltaic grid-connected inverter loss has been completed, and the design of the two-stage loss control scheduling method for the loss of the high-power photovoltaic grid-connected inverter has been realized.

## 5. Experimental Analysis

### 5.1. Experimental Environment

**Figure 3:**
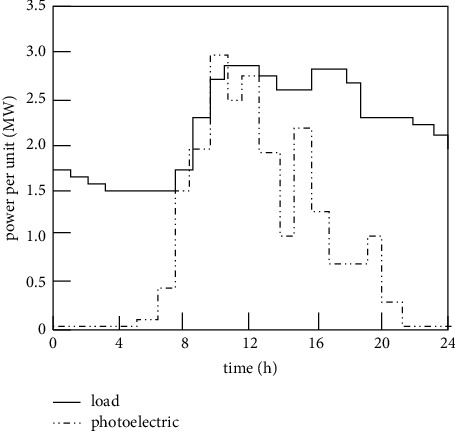
Daily operation curves of photovoltaic grid and load.

In order to verify the overall effectiveness of the intelligent control method of loss distribution balance of high-power photovoltaic grid-connected inverters, it is necessary to carry out relevant tests. An active distribution network is selected as the optimization object of the four methods. The distribution network is a combined electricity-heating type, the installed photovoltaic capacity is 1 MW, the rated capacity of the cogeneration unit is 2500 kW, the rated capacity of the electric energy storage is 2300 kWh, and the photovoltaic capacity is 2300 kWh. The output is fluctuating, and the peak load is taken as the power per unit value. The distributed power and daily load curves are shown in Figure 3.

According to Figure 3, the standard value of distribution network load is 2.95 MW and the PV unit value is 3.02 MW. When the design method is connected to the photovoltaic inverter, the DC voltage source and the controlled current source are, respectively, used to replace the front stage and the rear stage. The key parameters of the power circuit of the photovoltaic inverter are shown in Table 1.

Based on the key parameters of photovoltaic inverter in Table 1, the simulation results of intelligent control of loss distribution balance of high-power photovoltaic grid-connected inverter under the condition of severe uneven sunlight are studied by simulation experiment. The simulation time is 3 s, and the photovoltaic units keep the light intensity at 800 W/m^2^. The corresponding high-power photovoltaic grid-connected inverter output voltage, output current, and loss control effects are given. The antiresonance capability of the high-power photovoltaic grid-connected inverter is analyzed.

### 5.2. Analysis of Experimental Results

**Figure 4:**
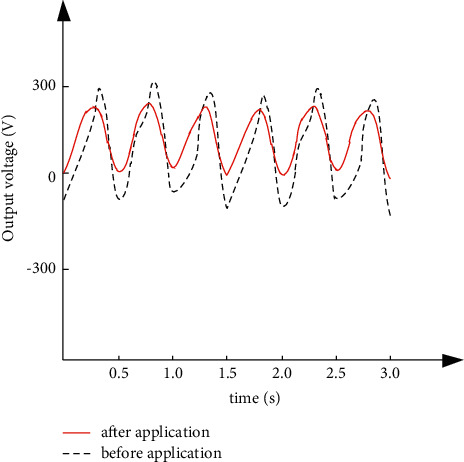
Waveform diagrams before and after the output voltage is applied.

**Figure 5:**
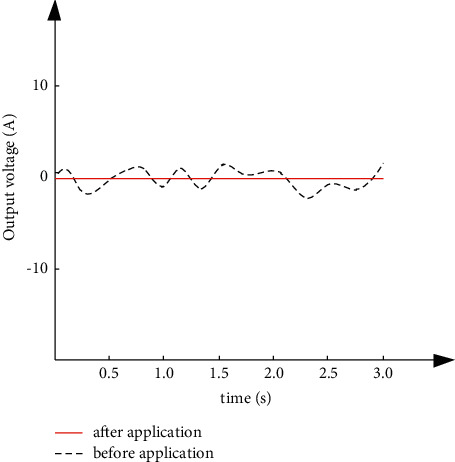
Waveform diagrams before and after the output current is applied.

According to the simulation parameter settings, test the waveforms of the output voltage and output current of the high-power photovoltaic grid-connected inverter before and after the application of the method in this paper, and test the regulation effect of the method in this paper in turn. The results are shown in Figures 4 and 5.

It can be seen from Figures 4 and 5 that, after the method in this paper regulates the joint operation of high-power photovoltaic grid-connected inverters in the power grid, the output voltage waveform can be optimized and the current fluctuation can be suppressed, which verifies the effectiveness of the method in this paper for loss control.

**Figure 6:**
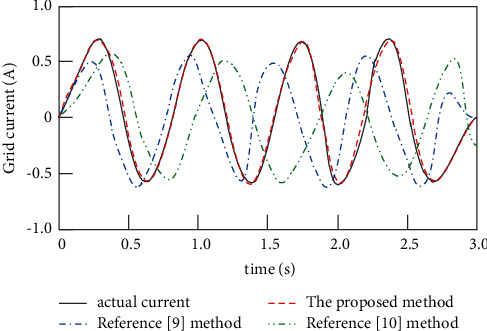
Grid current tracking results.

The proposed method, the method of [9], and the method of [10] are used to track the grid-connected current of the high-power photovoltaic grid-connected inverter, and the tracking results are shown in Figure 6.

Analysis of Figure 6 shows that, under the same environment, there are errors in the method of [9] and the method of [10] when tracking the incoming current, and the tracking results of the proposed method are basically consistent with the actual current waveform, indicating that the proposed method has better performance and tracking effect.

**Figure 7:**
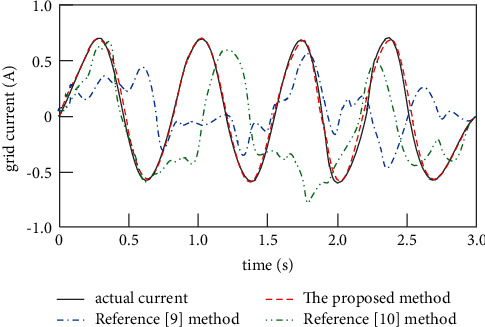
Grid current tracking results with changing grid-side point inductance.

Change the inductance value of the grid-side point, and continue to use the proposed method, the method of [9], and the method of [10] to track the incoming current in the above test environment, and the tracking results are shown in Figure 7.

Analysis of Figure 7 shows that when the grid-side inductance of the proposed method changes, the tracking results are still basically consistent with the actual grid-connected current, indicating that the proposed method has high robustness. Changing the grid-side inductance value can be regarded as an external disturbance, so the loss affecting the high-power photovoltaic grid-connected inverter was analyzed before the proposed method to control the grid-connected current. On this basis, a two-stage loss control method was designed to control the grid-connected current. Therefore, for the external disturbance in the control process, the proposed method shows good robustness.

**Figure 8:**
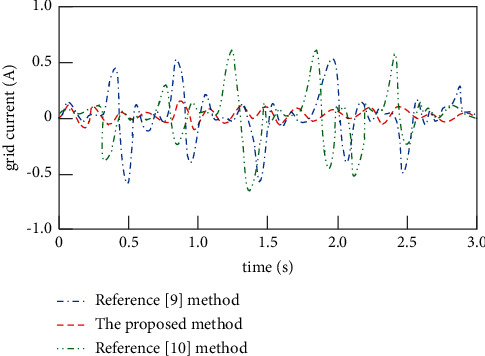
Antiresonance capabilities of different methods.

A trace disturbance signal of 1000 rad/s is introduced into the control process of the double closed-loop grid-connected current of the inverter, and the antiresonance capabilities of the proposed method, the method in [9], and the method in [10] are compared, as shown in Figure 8.

It can be seen from Figure 8 that, after the introduction of microdisturbance signal, the network access current control curve obtained by the proposed method is relatively smooth, and there is no resonance peak, indicating that the proposed method has strong antiresonance capability. However, after the introduction of microdisturbance signal, there are multiple resonance peaks in the network current control curve and the surface smoothness is low, which shows that the resonance peaks of microdisturbance signal noise cannot be eliminated when the methods in [9] and [10] are used to control the loss of high-power photovoltaic grid-connected inverter. Compared with the proposed method, the network current control effects of the methods in [9] and [10] are poor, with weak antiresonance capability.

To sum up, the output voltage waveform can be optimized and the current fluctuation can be suppressed at 0A after the joint operation regulation of high-power photovoltaic grid-connected inverters in the power grid. Under the same environment, the tracking results of the proposed method are basically consistent with the actual current waveform. When the network side inductance changes, the tracking result is still basically consistent with the actual network current. After introducing microdisturbance signal, the control curve of network access current obtained by the proposed method at 0A is relatively smooth, there is no resonance peak, and the proposed method has strong antiresonance capability.

## 6. Conclusion

The control of photovoltaic grid connection is a necessary condition for photovoltaic system power generation and a key technology related to the utilization of new energy. It is very important for the intelligence, efficiency, and popularization of microgrid in the future. This paper proposes an intelligent control method for the loss distribution balance of high-power photovoltaic grid-connected inverter. Based on the analysis of the loss distribution of high-power photovoltaic grid-connected inverter, a two-stage loss control objective function is proposed. In the first stage, the scheduling costs such as photovoltaic energy and fluctuating load are selected, and, in the second stage, the penalty costs of light abandonment and load loss are selected, which are limited by branch power flow and charging and discharging power of distribution network. A two-stage loss control model of distribution network is built and the optimal solution of the model is searched. Finally, through experiments, the correctness and feasibility of the loss analysis and loss control strategy of photovoltaic grid-connected inverter are verified, which reflects the effectiveness of this method for loss control, good tracking effect, strong robustness, and strong antiresonance capability.

## Figures and Tables

**Table 1:** Key parameters of PV inverter.

Parameter	Numerical value
Number of PVs installed in the node	8
Inverter rated capacity	4000 kW/h
Maximum charge and discharge power	450 kW
Inverter total capacity	4500 kW/h
Distribution grid voltage	220 V
Output filter inductor	7mH/0 Ω
Reactive power compensation range	−100∼300 kvar
DC bus capacitance	2500 uF
DC bus voltage	450 V
Distribution network frequency	60 Hz
Inverter remaining capacity threshold	6.0kVA
Operating frequency	40 kHz
DC boost inductor	5 mH

## Data Availability

The raw data supporting the conclusions of this article will be made available by the authors, without undue reservation.
